# Complement system contributes to modulate the infectivity of susceptible TcI strains of *Trypanosoma cruzi*

**DOI:** 10.1590/0074-02760170332

**Published:** 2018-02-19

**Authors:** Ruben D Arroyo-Olarte, Ignacio Martínez, Mayra Cruz-Rivera, Fela Mendlovic, Bertha Espinoza

**Affiliations:** 1 Universidad Nacional Autónoma de México, Instituto de Investigaciones Biomedicas, Departamento de Immunología, Ciudad de Mexico, Mexico; 2 Universidad Nacional Autónoma de México, Facultad de Medicina, Departamento de Microbiología y Parasitología, Ciudad de Mexico, Mexico; 3 Universidad Anahuac, Facultad de Ciencias de la Salud, Mexico Norte, Estado de Mexico, Mexico

**Keywords:** complement system, Trypanosoma cruzi, infection

## Abstract

**BACKGROUND:**

*Trypanosoma cruzi* is a protozoan parasite and an etiological agent of Chagas disease. There is a wide variability in the clinical outcome of its infection, ranging from asymptomatic individuals to those with chronic fatal mega syndromes. Both parasite and host factors, as well as their interplay, are thought to be involved in the process.

**OBJECTIVES:**

To evaluate the resistance to complement-mediated killing in two *T. cruzi* TcI strains with differential virulence and the subsequent effect on their infectivity in mammalian cells.

**METHODS:**

Tissue-culture derived trypomastigotes of both strains were incubated in guinea pig serum and subjected to flow cytometry in order to determine their viability and complement activations. Trypomastigotes were also incubated on host cells monolayers in the presence of serum, and infectivity was evaluated under different conditions of complement pathway inhibition. Relative expression of the main parasite-specific complement receptors between the two strains was assessed by quantitative real-time polymerase chain reaction.

**FINDINGS:**

In this work, we showed that two TcI strains, one with lower virulence (Ninoa) compared to the other (Qro), differ in their resistance to the lytic activity of complement system, hence causing a compromised ability of Ninoa strain to invade mammalian cells. These results correlate with the three-fold lower messenger RNA (mRNA) levels of complement regulatory protein (CRP), trypomastigote-decay acceleration factor (T-DAF), and complement C2 receptor inhibitor trispanning (CRIT) in Ninoa compared to those in Qro. On the other hand, calreticulin (CRT) mRNA and surface protein levels were higher in Ninoa strain and promoted its infectivity when the lectin pathway of the complement system was inhibited.

**MAIN CONCLUSIONS:**

This work suggests the complex interplay of CRP, T-DAF, CRIT, and CRT, and the diagnostic value of mRNA levels in the assessment of virulence potential of *T. cruzi* strains, particularly when dealing with isolates with similar genetic background.

The parasite *Trypanosoma cruzi* is the causative agent of Chagas disease, occurring in the American continent and spread by migration of infected people to other parts of the world. Eight million people are estimated to be infected and a further 100 million people are at risk (WHO 2017). *T. cruzi* is a trypanosomatid parasite that shows a high rate of genetic polymorphism and has been categorised into six discrete typing units (DTUs) based on diverse molecular markers (Zingales et al. 2009). Like other members of the Trypanosomatidae family, *T. cruzi* is a digenetic organism; its life cycle alternates between two different hosts: an invertebrate host (insects of the Reduviidae family, which act as vectors) and a vertebrate host (mainly wild and domestic mammals and humans) (de Souza 2002). The complement system is the primary defence mechanism and a mediator between host innate and adaptive immune responses that pathogens must efficiently overcome to establish an infection (Lambris et al. 2008). It has been earlier shown that metacyclic trypomastigotes of *T. cruzi* can indeed be killed by the complement system and that there is a wide variation in the degree of susceptibility or resistance among different *T. cruzi* strains, regardless of their genetic or host-origin background (Cestari & Ramírez 2010). Several complement receptors in *T. cruzi* have been identified to be responsible for the trypomastigote resistance to complement-mediated killing by inhibiting the formation of C3 convertases. These include complement regulatory protein (CRP), trypomastigote-decay accelerating factor (T-DAF), complement C2 receptor inhibitor trispanning (CRIT), and calreticulin (CRT) (Osorio et al. 2012).

The clinical outcome of Chagas disease is also influenced by the strain background, though not necessarily by their genetic background. We have previously shown that two Mexican *T. cruzi* strains, belonging to DTU I, display remarkably different pathogenesis in a murine model; the highly virulent Queretaro (Qro) strain causes 100% mortality in mice during the acute phase while the Ninoa strain does not result in mortality. In addition, infection with bloodstream trypomastigotes of Qro strain reaches a higher blood parasitaemia and induces extensive cardiac and skeletal muscle damage and more gastrointestinal inflammation than Ninoa strain (Espinoza et al. 2010, 2011). In the present work, we aim to study the mechanisms of resistance/susceptibility to complement-mediated killing for both these strains and understand the correlation with their infectivity in mammalian cells.

## MATERIALS AND METHODS

*Parasite strains and cell culture* - Mexican *T. cruzi* I Qro (TBAR/MX/0000/Queretaro) and Ninoa (MHOM/MX/1994/Ninoa) strains were used in this study (Espinoza et al. 2010). The Qro strain was isolated from the *Triatoma barberi* vector from the state of Queretaro in Central Mexico. The Ninoa strain was obtained from a human carrier in Oaxaca, a state in the southern Pacific coast of Mexico. Tissue culture trypomastigotes (TCT) of both strains were collected by centrifugation of the supernatant of previously infected Vero cell cultures at 800 × g at room temperature (25ºC) for 10 min, followed by incubation at 37ºC for 2 h, in order to allow TCT to move from the pellet into the supernatant. Thereafter, the supernatant was collected and the purified trypomastigotes were counted in a Neubauer chamber. Vero cells (ATCC CCL81™), were grown at 37ºC in Dulbecco’s minimum essential medium (DMEM) supplemented with 5% FBS, 100 µg/mL streptomycin, and 100 U/mL penicillin, in a humidified 5% CO_2_ atmosphere.

*Antibodies and sera* - Polyclonal goat anti-C3 antibody was obtained from Sigma-Aldrich (St. Louis, MO, USA); fluorescein isothiocyanate (FITC)-conjugated rabbit anti-goat antibody was from Santa Cruz Biotechnologies (Dallas, TX, USA). Goat IgG from goat serum was used as isotype control. Monoclonal mouse anti-C5b-9 IgG2a was from Santa Cruz Biotechnologies (Dallas, TX, USA); FITC-labelled anti-mouse IgG+IgM+IgA antibody was from Sigma Immunochemicals (St. Louis, MO, USA); mouse IgG2a was used as isotype control. Rabbit anti-GAPDH and HRP-labelled donkey anti-rabbit antibodies were from Sigma (St. Louis, MO, USA).

Guinea-pig serum (GPS) was used as complement source, as it has long been reported to have a higher cell lytic activity than that of most mammalian species (Hoet et al. 1954). GPS was obtained from blood that was allowed to coagulate at 0ºC followed by centrifugation (1000 × *g*, 10 min, 4ºC). Aliquots were stored at -70ºC until use. For control conditions without complement activity, GPS was heat-inactivated at 56ºC for 30 min.

To generate hyper immune serum against *Taenia solium* calreticulin, two mice (Balb/c) were immunised thrice, at one-week intervals, with 50 µg of recombinant *Taenia solium* calreticulin (*Ts*CRT) and complete Freund’s adjuvant (1:1) in the first immunisation, and incomplete Freund’s adjuvant in the second and third immunisations. At the end of the protocol, the mice were bled by cardiac puncture and serum was collected. Mice were maintained under pathogen-free conditions and provided with food and water ad libitum. Mouse polyclonal anti-*Tc*sHsp16 (small-heat shock protein 16) was used as an unrelated antibody control.

*Animals* - Male Hartley guinea pigs (weighing 200-300 g) were kindly donated by Dr Alberto Pizaña of the National Institute of Respiratory Diseases (Mexico City, Mexico). Animals were anaesthetised and sacrificed according to UNAM Animal Care guidelines (http://www.biomedicas.unam.mx/wp-content/pdf/intranet/reglamentos/codigo-etico-iibo.pdf?x21431) to obtain blood by cardiac puncture.

*Viability assays* - The LIVE/DEAD® Cell Viability kit was from Thermo Fisher Scientific (Waltham, MA, USA) and used according to the manufacturer’s instructions. Briefly, 1 × 10^6^ trypomastigotes/mL was incubated for 1 to 6 h in absence or presence of 50% GPS. Post incubation, the parasites were centrifuged at 3000 × *g* for 10 min to remove the supernatant, washed twice with PBS, and the parasite pellet from each treatment was re-suspended in 997 μL of PBS, 4 μL of a 50 mM solution of Calcein-AM (C-AM), and 1 μL of 2 mM ethidium homodimer (EthD-1). The samples were then incubated for 20 min at room temperature and immediately analysed using a flow cytometer (BD FACSCalibur flow cytometer), with a 530/30-nm filter (FL1-H) for C-AM (green fluorescence: living cells) and a 670 nm long-pass filter (FL3-H) for EthD-1 (red fluorescence: dead cells); 15,000 events per treatment were acquired. The data were analysed using FlowJo 7.3.2 software and expressed as the percentage of cells per population phenotype. The compensation was performed using live parasites in medium alone, stained only with C-AM, and dead parasites exposed to heat shock (65ºC for 10 min), stained with EthD-1. Three independent, by duplicated, experiment were performed.

*C3 and C5b-9 complement deposition assays* - To assess complement cascade activation, complement component C3 deposition assays were performed. Briefly, 0.5 × 10^6^ purified TCT were incubated with 50% GPS as complement source for 1 h at 37ºC. Parasites were then centrifuged (3,000 × g for 10 min at 4ºC) and washed twice with PBS. The parasite pellet was re-suspended in PBS/BSA 1% and incubated with polyclonal goat anti-C3 IgG (diluted 1:500) for 1 h at 0-4ºC, followed by centrifugation and washing with PBS. FITC-conjugated rabbit anti-goat IgG (diluted 1:1000) was then used as secondary antibody for 45 min at 0-4ºC and washed twice with PBS to remove any unbound antibody (Spiller 2000). Flow cytometric analysis was performed immediately in a BD FACSCalibur, with a 530/30-nm filter (FL1-H) for FITC, and plotted against cell size (forward-scatter, FSC-H), as 5% probability contours with outliers. To corroborate that the activation of complement cascades by *T. cruzi*, in presence of GPS induced membrane attack complex (MAC) deposition, an anti-C5b-9 mouse IgG2a (diluted 1:80) was used as primary antibody followed by a FITC-labelled anti-mouse IgG+IgM+IgA (diluted 1:400) as secondary antibody.

*Total RNA isolation* - Total RNA was isolated from 50 × 10^6^ purified TCT, derived from infected Vero-cell culture supernatants, using TRIzol (Thermo Fisher Scientific, Waltham, MA, USA), according to manufacturer’s instructions, followed by DNase I treatment. RNA integrity was analysed by 1% agarose gel electrophoresis in TBE buffer (40 mM Tris-Cl, 45 mM boric acid, 1 mM EDTA, pH 8), and visualised under UV light after ethidium bromide staining. Nucleic acid purity was assessed by the *A*_260 nm_/*A*_280nm_ ratio (acceptable > 1.8).

*Quantitative real-time polymerase chain reaction (qRT-PCR)* - DNase-treated total RNA (100 ng) was used as template for one-step quantitative RT-PCR with a Universal KAPA SYBR FAST One-Step qRT-PCR Kit (Kapa biosystems, Boston, MA, USA) in a Rotor Gene 6000 (Corbett Research, Australia) using the following gene-specific oligonucleotides: *T-DAF* (GenBank: XM_809679.1):- sense: 5′-CAGTTTGTCTTCTGTCGGGG-3′, anti-sense: 5′-GTTGTCACCTCCCCATGAAC-3′; *CRP* (GenBank: XM_811368.1):- sense: 5′-GCACTGCGAATGGATGGTGA-3′, anti-sense: 5′-CCGTTGCACTTTGGTTCTT-3′; *CRIT* (GenBank: AY464185.1):- sense: 5′-TCACAAGACAGCCAACTCACT-3′, anti-sense: 5′-ATGTGGAGGACATGAATCCGAG-3′; *CRT* (GenBank: AF107115.1):- sense: 5′-GCCGCCGACAACTCCTAC-3′, anti-sense: 5′-ATCCATCGTCTCCTCGTCCA-3′; *GAPDH* (GenBank: X52898.1):- sense: 5′- AGCATACAGGAGATCGACGC-3′, anti-sense: 5′-CGTAAATGGAGCTGCGGTTG-3′. The thermal program used was: 42ºC for 10 min (reverse transcription) and 95ºC for 3 min, followed by 45 cycles of: 95ºC for 5 sec, 60ºC for 20 sec, and 72ºC for 20 sec. A melting curve analysis (59ºC to 95ºC, 0.1ºC/sec) was performed immediately after qRT-PCR to confirm single peak-products and absence of primer-dimers. Additionally, the expected size of amplicons was confirmed by analysis of the real-time PCR products in 1% agarose gels in TBE, stained with ethidium bromide and visualised under UV light. In all cases *GAPDH* (glyceraldehyde 3-phosphate dehydrogenase) was used as reference gene, and target gene-expression levels were compared between Ninoa and Qro strains according to the following formula:

$$\mathrm{Relative}\;\mathrm{Expression}=\frac{{\left(\mathrm{Efficiency}{\;}_{target}\right)}^{\Delta \;CT\;target\;(\;averange\;of\;Qro-averange\;of\;Ninoa\;)}}{{\left(\mathrm{Efficiency}{\;}_{reference}\right)}^{\Delta \;CT\;reference\;(\;averange\;of\;Qro-averange\;of\;Ninoa\;)}}$$

Calculations were performed using REST software 2009, RG mode, with 10,000 permutations (Pfaffl et al. 2002), and PCR efficiencies calculated by Rotor-Gene 6000 software v.1.7.87.

*Host-cell invasion assays* - Three thousand Vero cells per well were seeded on 21-well-PTFE printed slides (Electron Microscopy Sciences, Hatfield, PA, USA) in DMEM supplemented with 5% FBS and incubated for 12 h at 37ºC in a humidified 5% CO_2_ atmosphere to allow cell adhesion. Purified TCT from previously infected Vero cell culture were then added at a multiplicity of infection (MOI) of 10 parasites:1 host cell for both strains and incubated for 6 h at 37ºC, 5% CO_2_. Experiments ranging from 15 to 120 min were also performed at an MOI of five parasites: 1 host cell. Depending on the experimental conditions, heat-inactivated (control) or freshly-thawed 50% GPS were incubated, with or without sugars [mannose plus N-acetyl glucosamine (Man+NAcGlc) or galactose (Gal), 100 mM each] and/or antibodies (polyclonal anti-*Ts*CRT or anti-*Tc*sHsp16) before being used for the infection experiments. Glass slides were then washed twice in PBS, and fixed in 100% methanol for 5-10 min. DAPI (4’,6-diamidino-2-phenylindole) was used at 50 nM for 20 min at room temperature for nuclear staining. Slides were visualised under a Nikon epifluorescence microscope and at least 200 cells were counted to determine the percentage of infected cells and number of intracellular parasites.

*Lectin-probed western blot analysis* - To study glycoprotein composition of both strains, lectin blots were performed using the DIG Glycan Differentiation Kit (Roche, Switzerland) according to the manufacturer’s instructions. Briefly, protein extracts from Ninoa and Qro strains were prepared following a TRIzol-modified protocol (Simões et al. 2013). Proteins (10-15 µg per lane) were separated by 8% SDS-PAGE and transferred to nitrocellulose membranes. Probing was conducted with digoxigenin-labelled *Galanthus nivalis* lectin (GNL) or *Datura stramonium* agglutinin (DSA), followed by incubation with anti-digoxigenin coupled with alkaline phosphatase. A NBT/BCIP mix was used as a revealing solution. Rabbit anti-GAPDH followed by HRP-labelled donkey anti-rabbit antibody was used as loading control. In this case, a 3,3’-diaminobenzidine/H_2_O_2_ solution was used as substrate and revealing solution.

*Analysis of total and surface TcCRT protein expression* - To evaluate total calreticulin expression in Ninoa and Qro TCT, 10–15 µg protein extracts were subjected to 8-12% SDS-PAGE, transferred to nitrocellulose membranes, and probed with a polyclonal mouse anti-*Taenia solium* calreticulin IgG. Rabbit anti-GAPDH was used as loading control, followed by HRP-goat anti-rabbit IgG (Thermo Fisher Scientific, Waltham, MA, USA) as secondary antibody, and staining with 3,3’-diaminobenzidine/H_2_O_2_ solution. On the other hand, to examine the surface expression of TcCRT, Ninoa and Qro TCT were subjected to flow cytometric analysis, similarly as explained above, for complement deposition assays. Briefly, 0.5 x 10^6^ TCT were incubated with anti-*T. solium* calreticulin IgG as primary antibody (1:500, 1 h, 0ºC) followed by staining with FITC-labelled-anti mouse IgA+IgG+IgM (1:1000, 45 min, 0ºC) as secondary antibody. Acquisition was done immediately in a BD FACSCalibur with a 530/30-nm filter (FL1-H) for FITC, and plotted against cell size (forward-scatter, FSC-H), as 5% probability contours with outliers. Control experiments were performed with secondary antibody alone.

*Data analyses* - Statistical analyses were performed using the Prism software v. 6.01 (GraphPad). All experiments were done in triplicate, unless stated otherwise. Analysis of variance (ANOVA) was followed by Bonferroni post-hoc tests for significance assessment (p < 0.05).

## RESULTS

**Fig. 1:**
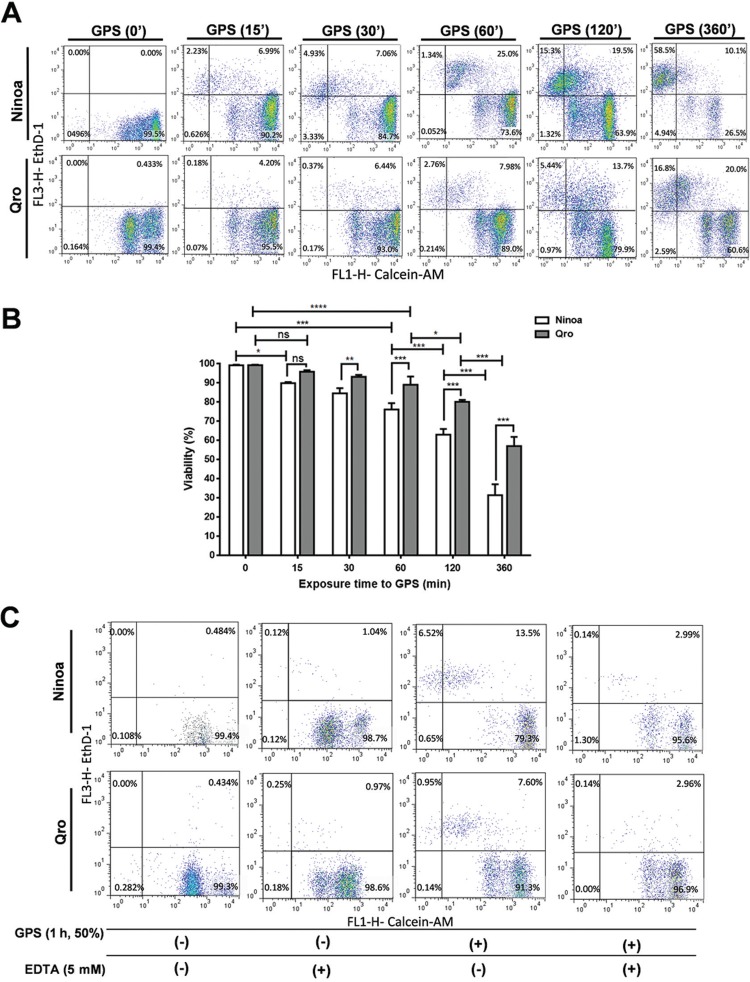
: Ninoa and Qro tissue culture trypomastigotes show differential resistance to complement-mediated killing. (A) Cell viability assays by flow cytometry. Tissue culture trypomastigotes (TCT) of Ninoa and Qro strains were incubated in Dulbecco’s minimum essential medium (DMEM) plus 50% fresh guinea-pig serum (GPS). After treatment over the indicated times, the cells were collected and subjected to Calcein (FL1-H)/Ethidium homodimer (FL3-H) double-staining for flow cytometry. The lower right quadrant (Calcein+/Eth-H-) was considered as intact viable cells; the sum of upper-right and upper-left quadrants (Eth-H+) was considered as dead, cell-membrane damaged cells. (B) Summary of flow cytometry experiments show the percentage of viable trypomastigotes of both strains after the indicated incubations with GPS. Data are means ± standard deviation (SD) (N = 3, **p < 0.01, ***p < 0.001). (C) Flow cytometry plots show living or dead Ninoa and Qro TCT after incubation with guinea pig serum alone, or in combination with EDTA to chelate divalent cations (Ca2+, Mg2+) for inhibition of all complement system pathways.

*Qro strain is more resistant than Ninoa to complement-mediated killing in a time-dependent manner* - When fresh Ninoa and Qro tissue culture trypomastigotes (TCT) were incubated with non-immune guinea pig serum (GPS) as source of complement, there was a time-dependent death as shown by our viability assays (Fig. 1A). However, the kinetics of serum-induced lysis was significantly faster for Ninoa than Qro TCT after only 30 min up to 6 h of serum incubation (Fig. 1B). To corroborate that the lytic activity of serum was dependent on the complement system activity, Ninoa and Qro TCT were also incubated in presence of EDTA and GPS as negative controls (Fig. 1C). These results show a higher susceptibility of Ninoa TCT to complement-mediated lysis compared to that of the more virulent Qro strain.

**Fig. 2:**
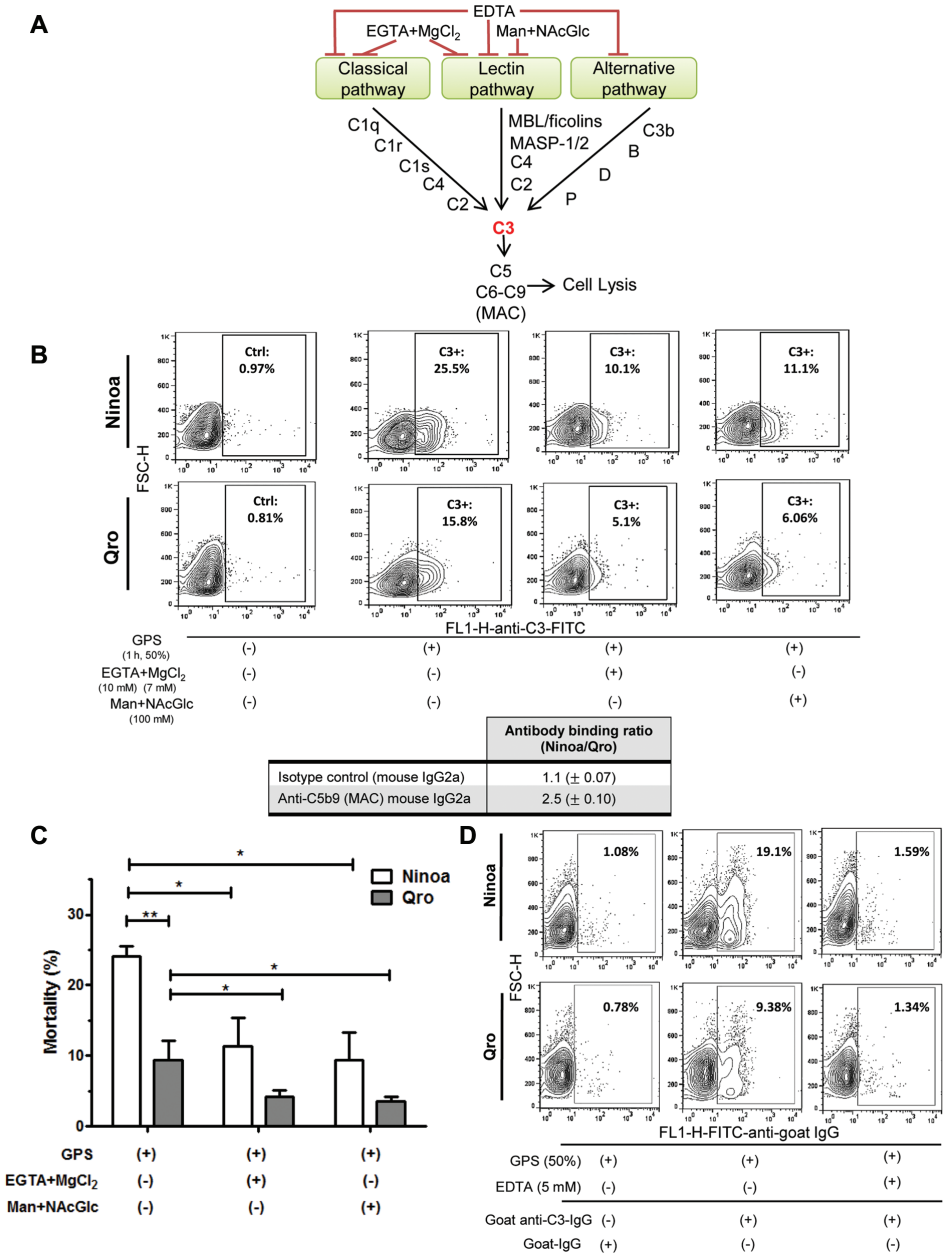
: Ninoa tissue culture trypomastigotes (TCT) activate alternative and lectin pathways of the complement system more efficiently than Qro TCT. (A) Scheme showing complement-system activation pathways; all three complement pathways converge in the activation of C3 factor by C3 convertases. B. Flow cytometry plots show C3-positive (rectangle) trypomastigotes of Ninoa and Qro strains after 1-hour incubation in the presence or absence of guinea-pig serum (GPS) as complement source. TCT were also co-treated with EGTA+Mg2+ to inhibit classical and lectin pathways, or with sugars (mannose plus N-acetyl glucosamine) to selectively inhibit the lectin pathway. To corroborate membrane attack complex deposition, assays in presence of GPS for 1 h were performed with an anti-C5b-9 antibody; the ratio of antibody binding between Ninoa and Qro is shown. Mouse IgG2a was used as isotype control. Results are mean ± standard deviation (SD) (N = 2). (C) Summary of cell viability assays show changes in the percentage of dead cells (ethidium-homodimer positive, that is, percentage of cells in the upper left and upper right quadrants as assessed in Fig. 1A), for the indicated conditions. Data are means ± SD (N = 3, *p < 0.05, **p < 0.01). (D) Representative flow cytometry plots (N = 2) after 1 h incubation of Ninoa and Qro trypomastigotes with GPS alone or with EDTA to inhibit complement system; parasites were subjected to indirect immunostaining with either goat IgG (from goat serum) as isotype control or anti-C3 as primary antibodies, and fluorescein isothiocyanate (FITC) conjugated donkey anti-goat IgG as secondary antibody. C3-positive parasites were scored in the shown gate for FITC staining.

*Ninoa TCT activate the alternative and lectin pathways of the complement system more efficiently* - For a detailed investigation of the complement pathways activated by TCT, experiments with anti-C3 antibody were performed after pre-incubation with GPS. As shown in Fig. 2A, all three complement pathways converge in activation and deposition of C3 before pathogen lysis by membrane attack complex (MAC). We found more C3 binding to Ninoa than to Qro TCT (25.5% vs. 15.8%) after 1 h incubation with GPS (Fig. 2B). A similar difference in MAC deposition was seen with a 2.5-fold increase in anti-C5b-9 binding to Ninoa compared to Qro TCT in presence of GPS (50%, 1 h, Fig. 2B). When C3 deposition was analysed in presence of EGTA+MgCl_2_ to block the Ca^2+^-dependent classical and lectin pathways, there was a remarkable reduction of C3 binding to both strains, (25.5→10.1% for Ninoa vs. 15.8→5.1% for Qro). To further discriminate the contribution of classical and lectin pathways, trypomastigotes were also incubated with sugars, like Man+NAcGlc, to specifically inhibit mannose-binding lectins (MBLs) and ficolins, which are the initiator molecules of the lectin pathway (Fig. 2B). Binding of C3 was only about 1% higher than that found after EGTA+MgCl_2_ treatment (11.1% for Ninoa vs. 6.06% for Qro), indicating a minimal activation of the classical pathway by both strains when using non-immune serum.

Viability assays, under different conditions, to block specific complement-system activation pathways were also performed (Fig. 2C). Inhibition of lectin pathway alone (Man+NAcGlc) caused about 2.5-fold decrease in complement mediated-killing for both strains (24→9.4% for Ninoa vs. 9.3→3.6% for Qro), while simultaneous inhibition of the classical pathway (EGTA+MgCl_2_) caused no further change. On the other hand, mortality induced by the alternative pathway (EGTA+MgCl_2_) in Ninoa was higher than that in Qro trypomastigotes (11.4 vs 4.2%). However, the difference in sensitivity to the alternative pathway between Ninoa and Qro (7.2%) contributes to only half the difference in complement-mediated killing observed in control treatment (14.7%), thereby hinting at a higher resistance of Qro to the lectin pathway (Fig. 2C). Control isotype experiments were performed using goat IgG as primary antibody and rabbit FITC-anti-goat-IgG as secondary antibody after pre-incubation of parasites with GPS; less than 1% FITC-positive cells were observed. Similarly, when serum was treated with EDTA to block all complement pathways, the amount of FITC-labelled cells (when using anti-C3 as primary antibody) was below 1.5% for both strains (Fig. 2D).

**Fig. 3:**
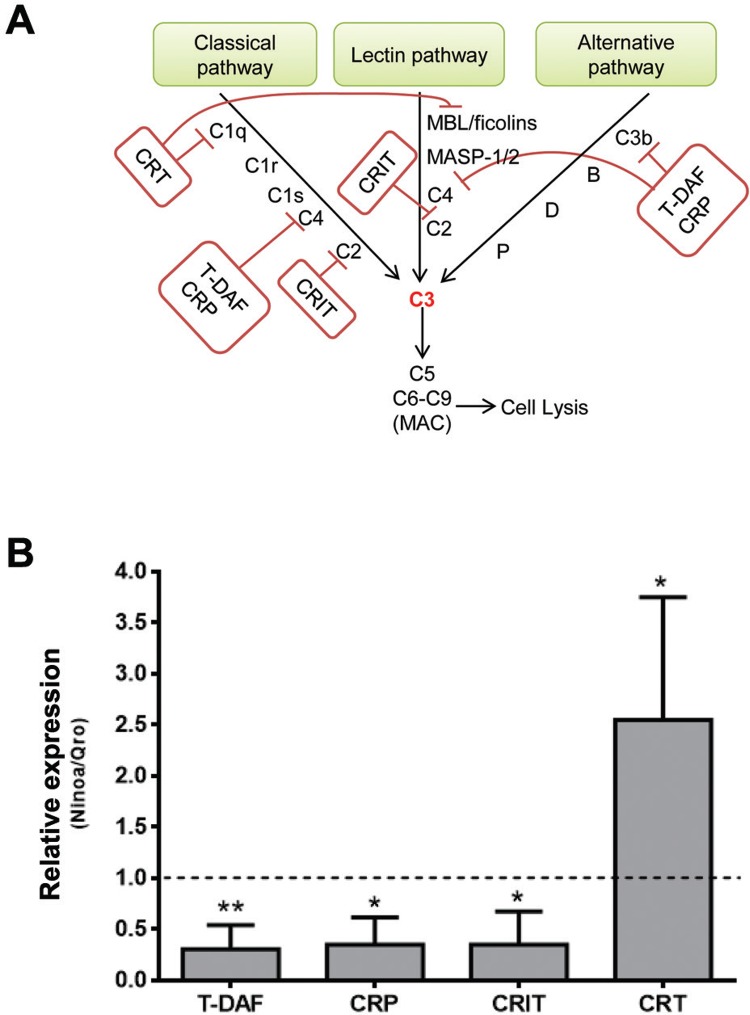
: Ninoa tissue culture trypomastigotes (TCT) display lower mRNA levels of the proteins involved in complement evasion such as complement regulatory protein (CRP), trypomastigote-decay acceleration factor (T-DAF), and complement C2 receptor inhibitor trispanning (CRIT), but not calreticulin (CRT). (A) Summary of the main complement-system evasion strategies of *Trypanosoma cruzi*. (B) Quantitative real-time polymerase chain reaction (qRT-PCR) analysis of mRNAs for CRP, T-DAF, CRIT and CRT in Ninoa TCT compared to those in Qro. Data were normalised by the amount of GAPDH mRNA, expressed relative to the corresponding value for Qro strain as control population, and are means ± SEM (N = 4, *p < 0.05, **p < 0.01).

*Susceptibility to complement-mediated lysis correlates with lower mRNA levels of CRP, T-DAF, and CRIT in Ninoa strain* - In order to correlate the differential sensitivity to complement-mediated lysis with the expression of complement-inhibiting proteins in the two strains, we analysed the mRNA levels of known inhibitory proteins (CRP, T-DAF, and CRIT) of the complement system in *T. cruzi* (Fig. 3A) by quantitative RT-PCR. About three-fold lower expression levels for all three complement-inhibitory proteins were found in Ninoa compared to those in Qro TCT, suggesting more efficient anti-complement defence machinery for the latter (Fig. 3B). Interestingly, CRT, a C1q-binding protein involved in the resistance to classical pathway and also able to bind to mannose-binding lectins (MBL) in the lectin pathway of the complement system, showed about 2.5-fold higher mRNA levels in Ninoa compared to that in Qro TCT.

**Fig. 4:**
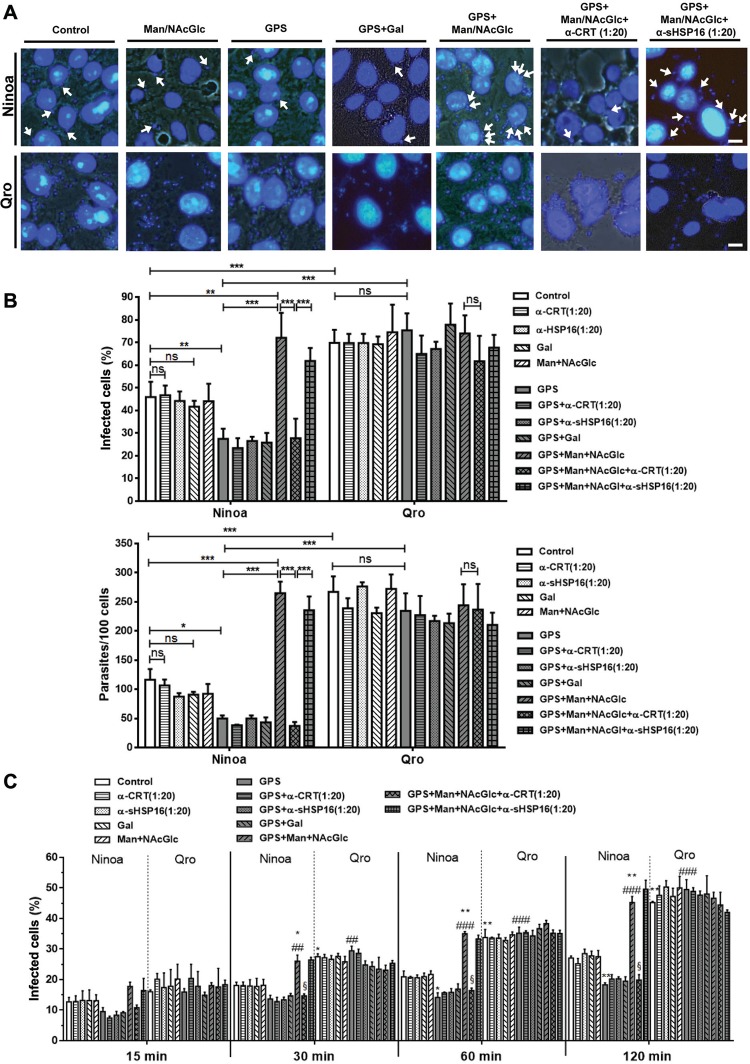
: mammalian cell invasion of Ninoa, but not Qro tissue culture trypomastigotes (TCT), is modulated by the complement system. (A) Fluorescence micrographs of bright-field and DAPI nuclear staining show intracellular parasites (white arrows) after co-incubation of Ninoa or Qro TCT with Vero cells in presence of 50% guinea-pig serum (GPS) alone, or along with sugars (galactose or mannose plus N-acetyl-glucosamine, 100 mM each) to inhibit the lectin pathway of the complement system for 6 h at 37ºC, or GPS plus sugars and anti-calreticulin (CRT) anti-serum (diluted 1:20). As controls, parasites and cells (10:1) were incubated with Dulbecco’s minimum essential medium (DMEM) plus 50% heat-inactivated GPS, or heat-inactivated GPS plus sugars, or plus anti-sHSP16 anti-serum (diluted 1:20). Scale bar = 10 µm. (B) Percentage of infected cells and number of intracellular parasites in the above-mentioned conditions are depicted. At least 200 cells were counted in all cases. Data are means ± standard deviation (SD) (N = 6, *p < 0.05, **p < 0.01, ***p < 0.001). (C) Percentage of infected cells in the annotated conditions for Ninoa and Qro TCT from 15 min up to 120 min of infection (five parasites: 1 Vero cell). At least 200 cells were counted in all cases. Data are means ± SD (N = 3; *p < 0.05, **p < 0.01, ***p < 0.001 compared to Ninoa control with heat-inactivated serum; #*p* < 0.05, ##p < 0.01, ###p < 0.001 compared to Ninoa with GPS; §p < 0.05, compared to Ninoa with GPS+Man+NAcGlc).

*Invasion of mammalian cells is regulated by the complement system for Ninoa, but not for Qro trypomastigotes* - To correlate the differential sensitivity to the complement system with the degree of virulence of both strains, invasion assays in presence of GPS for 6 h (MOI 10) were performed as shown by microscopy (Fig. 4A). We found that both, the percentage of infected cells, as well as the number of intracellular amastigotes (Fig. 4B), were significantly decreased in presence of normal GPS compared to the control (heat-inactivated serum) for Ninoa, though not so for the Qro strain, whose infectivity remained unaltered. However, the percentage of cells infected by Ninoa was always lower compared to that by Qro, even under control conditions (1.5-fold), the former being further decreased in presence of GPS (2.5-fold). A similar result was obtained regarding the number of intracellular amastigotes (2.5-fold lower for control; 5-fold lower for normal GPS). These results indicate that although Ninoa TCT were intrinsically less virulent than Qro, the difference in infectivity is enhanced by their lower resistance to complement-mediated lysis at physiological serum concentration. On the other hand, when the lectin pathway was inhibited by co-incubation of parasites and host cells with serum and Man+NAcGlc, the infectivity of Ninoa strain was greatly enhanced compared to that in either GPS alone or control conditions, and achieved similar values as in Qro strain, which remained unaffected in all the cases (Fig. 4B-C). This effect was, however, not observed when incubated with the sugars alone, and was fully inhibited by addition of anti-calreticulin polyclonal antibody (dilution 1:20). The anti-CRT antibody showed no significant effect when Man+NAcGlc were absent. A mouse polyclonal anti-sHSP16 antibody (1:20) was used as an unrelated antibody control that did not affect invasion capacity in any case. Addition of galactose (Gal, 100 mM), as an unrelated sugar control, caused no significant effect on the invasion of both strains.

When invasion was analysed for shorter durations at a lower parasite:host cell ratio (5:1), a significant decrease in infected cells for Ninoa strain was observed after 30-min incubation with GPS compared to that for Qro strain. There was also a significant reduction in host-cell infection for Ninoa strain after 60-min incubation with GPS, when compared to the control condition with heat-inactivated serum. Co-incubation with GPS and Man+NAcGlc (but not Gal) caused a significant increase in invasion for the Ninoa strain, from 30 min up to 120 min of infection, which was specifically inhibited by anti-CRT antibody (Fig. 4C).

**Fig. 5:**
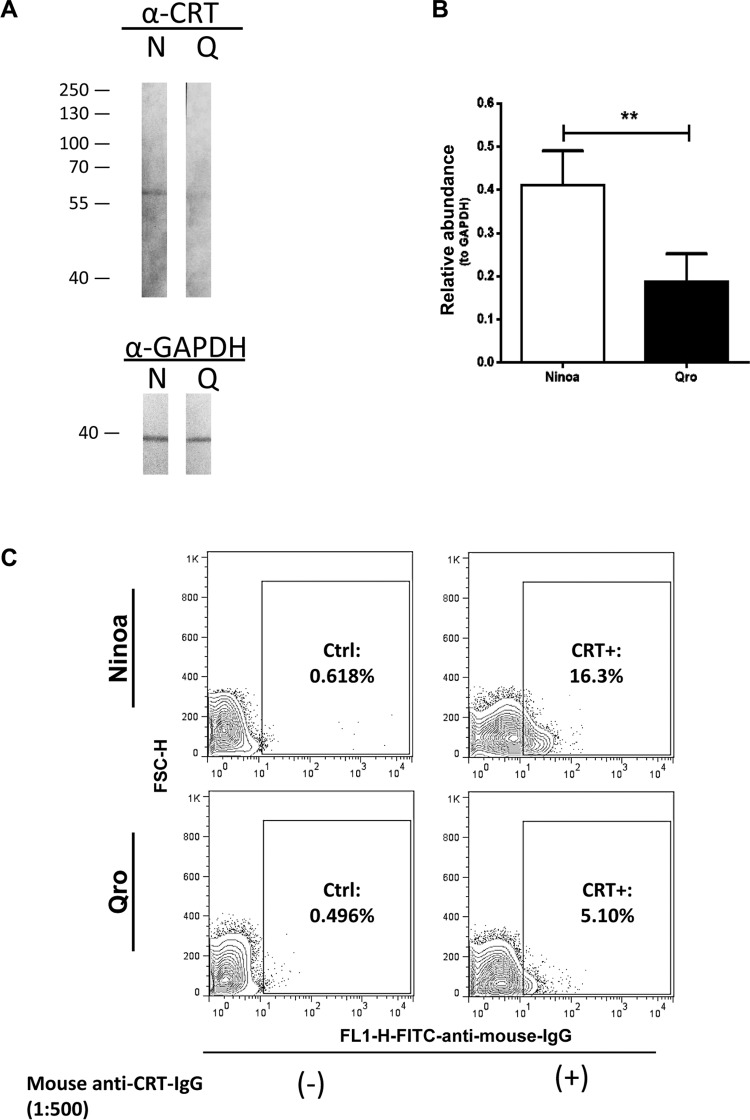
: Ninoa tissue culture trypomastigotes (TCT) express higher levels of total and surface calreticulin (CRT). (A) Representative immunoblot image showing calreticulin (TcCRT) in total protein extracts from Ninoa and Qro TCT, probed with anti-CRT antibody; lower panel shows anti-GAPDH blot used as loading control. (B) Relative densitometric analysis of TcCRT protein expression. Data were normalised to the corresponding GAPDH band, and are means ± standard deviation (SD). (N = 3, *p < 0.05, **p < 0.01). (C) Representative flow cytometry plots (N = 2) of Ninoa and Qro trypomastigotes for surface CRT. Parasites were subjected to indirect immunostaining with either mouse anti-CRT as primary antibody and fluorescein isothiocyanate (FITC) conjugated rabbit anti-mouse IgG as secondary antibody, or with secondary antibody alone as background control. CRT-positive parasites were scored in the shown gate for FITC staining.

*Ninoa trypomastigotes express higher levels of calreticulin on cell surface than Qro trypomastigotes* - To further explore possible reasons for the unexpected enhancement of Ninoa infectivity in presence of serum plus Man+NAcGlc and the corresponding blocking by anti-CRT antibodies, western blots were performed to evaluate the CRT protein levels in TCT of both strains. As seen in Fig. 5A-B, Ninoa TCT showed significantly higher expression level (approximately two-fold) of the 55 kDa band that corresponds to CRT as compared to the Qro strain. The expected molecular weight for *T. cruzi* CRT is about 45 kDa (Aguillon et al. 1995); the observed 55 kDa band in our blots may be explained by a variable electrophoretic mobility of calreticulin due to its high relative charge, as reported previously (Michalak et al. 1992). In order to interact with the complement system components, CRT must be translocated to the parasite cell surface; therefore, flow-cytometry assays with anti-CRT antibody were performed on non-permeabilised TCT of both strains (Fig. 5C). About three-fold higher amount of surface CRT-positive Ninoa TCT were detected compared to that in case of Qro (16.3% vs 5.10%), correlating with the up-regulation of CRT expression in Ninoa TCT.

**Fig. 6:**
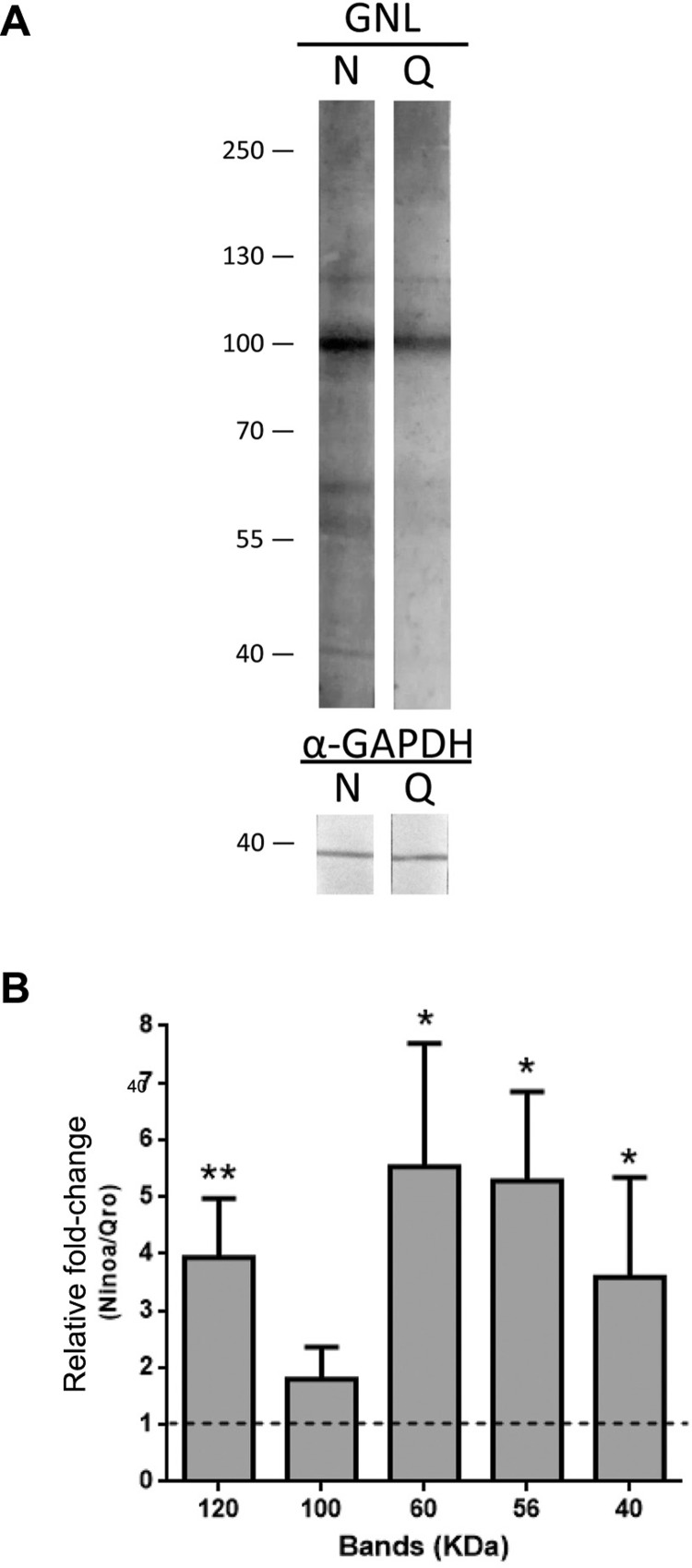
: lectin-probed western blots show a higher expression of mannose-containing glycoproteins in Ninoa than in Qro tissue culture trypomastigotes (TCT). (A) Representative western blot image (N = 3), showing mannose-positive glycoproteins in Ninoa and Qro TCT total protein extracts, probed with *Galanthus nivalis* lectin (GNL); GAPDH was used as loading control. (B) Relative densitometric analysis of the main mannose-positive glycoproteins. Data were normalised by the intensity of anti-GAPDH band, and expressed as a ratio to the corresponding value for Qro strain; data are means ± standard deviation (SD) (N = 3, *p < 0.05, **p < 0.01).

*Lectin binding to glycoproteins of Ninoa and Qro trypomastigotes* - In order to establish a definitive importance of the lectin pathway in the regulation of complement-mediated killing and host-cell invasion, particularly for Ninoa strain, we analysed the pattern of sugar-containing glycoproteins, the main target of MBLs, in both strains (Fig. 6A). Western blot with *Galanthus nivalis* lectin (GNL), specifically recognising mannose-containing glycoproteins, showed that Ninoa strain has significantly higher levels of mannose-glycoproteins with molecular weights about 120, 60, 56, and 40 kDa (Fig. 6B). No difference between the strains was found regarding NAcGlc containing glycoproteins when using specific *Datura stramonium* agglutinin (data not shown).

## DISCUSSION

The complement system is the main arm of innate immunity and the first obstacle for *T. cruzi* to establish an infection, as exemplified in birds, whose complement system is fully lytic against all life-stages of *T. cruzi* and keeps them refractory to infection (Kierszenbaum et al. 1976). It was long believed that virulent forms of *T. cruzi* are entirely resistant to mammalian complement system, and this concept has been applied for the experimental isolation of the different stages (Nogueira et al. 1975). A recent study showed a remarkable strain-specific variability concerning complement-system sensitivity of metacyclic trypomastigotes (MT) of *T. cruzi* (Cestari & Ramírez 2010). Despite being very similar stages, MT and bloodstream trypomastigotes differ in the key aspects of their invasion mechanism and exposure to complement system (Cortez et al. 2016). MT are released in the faeces or urine of the insect vector after a blood meal and are the first to enter the mammalian host through its bite. Poor parasite migration to surrounding tissues or draining lymph nodes and the evidence of parasite proliferation at the site of infection suggest that, the parasite invades tissues, but not immune cells, immediately after the initial infection (Padilla et al. 2009). Once inside, MT differentiate into amastigotes, which after intracellular replication, transform back to highly motile bloodstream trypomastigotes and trigger host cell rupture. By then, *T. cruzi* reaches the mammalian bloodstream and becomes a target of the complement pathways (Cardoso et al. 2016). In the present work, we analysed the complement sensitivity of TCT (equivalent to blood trypomastigotes) of two *T. cruzi* strains (DTU I) with differential virulence in a rodent model (Espinoza et al. 2010).

Our results demonstrate that the more virulent Qro strain TCT show higher resistance to complement-mediated killing compared to the Ninoa strain, in a time-dependent fashion, up to 6 h of incubation (Fig. 1A-B). We suggest that the higher resistance to complement lytic activity would curtail parasitaemia control and allow Qro trypomastigotes to expand more efficiently throughout the bloodstream to invade more tissues. On the other hand, Ninoa TCT experience significant complement-mediated killing right after 15 min of incubation with GPS, compared to 1-h incubation for Qro TCTs. This correlates with the lower number of parasitised cells for Ninoa infection, in presence of GPS, after 30 min of incubation (Fig. 4C).

Our analysis of C3 deposition, as a common step of all complement pathways (Fig. 2A), also shows a higher activation of the complement system by Ninoa than Qro TCT. There was an increased C5b-9 deposition on Ninoa TCTs (Fig. 2B), which ultimately leads to pathogen lysis (Lambris et al. 2008). When Ca^2+^, but not Mg^2+^, was chelated to block only the activation of the classical and lectin pathways (Cestari & Ramírez 2010), there was a drastic drop in C3 deposition for both strains, although not completely abolished, suggesting that complement activation via the alternative pathway is limited and less efficient. Inhibition of lectin pathway initiators MBLs and ficolins, by co-incubation with mannose and N-acetyl-glucosamine (Evans-Osses et al. 2014), caused similar decrease in C3 deposition, as well as in complement-induced mortality in both strains, indicating not only the pivotal role of lectin pathway but also, as shown earlier with other strains, the minimal contribution of the classical pathway when using non-immune serum or during acute infection (Cestari & Ramírez 2010, Cardoso et al. 2016). There are several strategies used by *T. cruzi* trypomastigotes to evade the complement system. The most common mechanism is the expression of complement receptors that block the assembly of C3 convertases (Cardoso et al. 2016). Among these receptors, CRP and T-DAF exhibit similarities with the human DAF protein. Both are able to inhibit the assembly of C3 convertases in all three pathways by binding to C4b and C3b (Tambourgi et al. 1993, , Norris 1998). A recent study has also demonstrated a correlation between CRP protein level and strain virulence *in vivo* (Henrique et al. 2016). Our qRT-PCR assay showed a three-fold lower mRNA expression for T-DAF, CRP, as well as for CRIT, a C2-binding protein and hence an inhibitor of classical and lectin pathways (Cestari et al. 2008), in the Ninoa strain. These results clearly correlate with the lower resistance of the Ninoa strain to complement-mediated killing. However, this was not the case for *T. cruzi* calreticulin (TcCRT), an important inhibitor of the initial phases of classical and lectin pathways by sequestering C1q (Valck et al. 2010) and binding to MBL/ficolins (Sosoniuk et al. 2014), respectively. TcCRT mRNA level was found to be up-regulated about 2.5-fold in Ninoa compared to Qro TCT. Taken together, these results suggest the importance of simultaneously analysing the expression profile of several complement receptors to correlate with the natural resistance or susceptibility of infective forms of *T. cruzi* isolates to complement-mediated lysis.

The effect of differential sensitivity to complement-mediated killing correlated with the infectivity of Ninoa and Qro TCT. Our 6-h long invasion assays showed intrinsic differential infectivity, as evidenced by an infection percentage of 70% for Qro vs. 45% for Ninoa, as well as a 2.5-fold lesser number of intracellular Ninoa parasites (Fig. 4B) in control conditions without complement activity. This fact suggests differential expression of other virulence factors, potentially regulating parasite invasion between both strains, e.g. cruzipain, gp82, gp90, oligopeptidase B, etc (Osorio et al. 2012). Addition of normal GPS induced a further 35-45% decrease in both invasion parameters for Ninoa, whereas there was no effect on Qro strain. This result correlates with the slower kinetics of complement-mediated killing of Qro TCT (Fig. 1B) and also to the faster invasion rate of Qro TCT, associated with high levels of infection (approximately 50% infected cells) after only 2 h (Fig. 4C). This rapid invasion rate would allow Qro TCT to escape the complement-mediated killing observed after prolonged exposures to serum (Fig. 1B). Intriguingly, when the lectin pathway of the complement system was specifically inhibited with mannose and N-acetyl glucosamine, Ninoa infectivity was not only recovered, but potentiated to similar values as that of Qro strain, which remained unaffected. TcCRT has been reported as an efficient complement-inhibitory protein and a potent virulence properties favouring the invasion of *T. cruzi* by coating the parasite with inactive C1q, which can still work as an apoptotic signal to trigger phagocytosis by macrophages. It also favours the attachment and invasion of host-cell membrane by binding to host calreticulin, as has been seen in the colonisation of placental tissue (Castillo et al. 2013), and more recently, using TcCRT-coated epimastigotes invading mammalian cells (Sosoniuk-Roche et al. 2017). We propose that in the GPS plus Man+NAcGlc condition, not only are Ninoa TCT free of the lytic activity of the lectin pathway, which is a major contributor of complement activation and complement-mediated killing (Fig. 2B-C), but also that the higher levels of TcCRT mRNA (Fig. 3B) and protein (Fig. 5A-C) allow them to sequester more C1q and enter the host cell via binding to host calreticulin. This possibility, however, requires further research. On the other hand, when polyclonal anti-CRT antibodies were added in presence of sugars and GPS, the infectivity of Ninoa was strongly reduced to similar values as in the GPS alone condition. The anti-CRT antibody used in our invasion assays is polyclonal, and it may also block the binding of host calreticulin to the C1q-TcCRT dimer. Besides, we suggest that binding of calreticulin to MBLs instead of C1q (under GPS condition), is of similar action as that to the anti-CRT antibody, and therefore similar values are observed under both conditions in Ninoa strain.

The ability to produce anti-TcCRT antibodies is associated with the control of parasitaemia and survival during acute infection in congenic mice (Aguillon et al. 1995). We suggest that the higher levels of TcCRT in the Ninoa strain would render it as a better immunogen than Qro TCT, thereby enabling the host anti-TcCRT antibodies to contribute to an efficient control parasitaemia and improve host survival, which does not happen for the highly virulent Qro strain. In fact, the isotype profile of immunoglobulins induced by both strains is also very different, including a major 45-50 kDa antigen (similar to the molecular weight of TcCRT), recognised only by the sera of Ninoa-infected mice, during both acute and chronic infection (Espinoza et al. 2010). TcCRT is a major immunogen during chronic Chagas disease and has been proposed to be a cause of auto-immunity, secondary to the production of cross-reactive antibodies to host calreticulin, once acute infection is controlled (Ribeiro et al. 2009). This may also contribute to the reported inflammatory response found in Ninoa-infected mice in diverse tissues, despite a rather effective control of parasitaemia, particularly during the chronic phase (Espinoza et al. 2010, 2011).

Finally, we performed lectin blots to observe parasite glycoproteins as possible targets of the lectin pathway in both strains (Fig. 6A-B). We found that Ninoa strain expressed significantly higher levels of glycoproteins with mannose residues of different molecular weights, which are potential MBL targets for lectin pathway activation (Luz et al. 2016). This would render Ninoa TCT more easily recognisable by the host MBLs and hence explain their higher activation of the complement system compared to that in the Qro strain.

Despite their common genetic background, both TcI strains seem to exert different strategies to cope with the complement system during acute infection. Qro strain associates with higher levels of three potent complement inhibitory proteins, CRP, T-DAF, and CRIT, and is able to compromise all three complement pathways. Furthermore, Qro TCT also express lower levels of PAMPs, such as mannose-containing glycoproteins, to reduce the activation of the complement cascades. On the other hand, Ninoa strain mainly relies on an enhanced expression of TcCRT to cope with complement system activation, particularly targeting the classical and lectin pathways. Calreticulin would also favour the intracellular invasion of Ninoa TCT by sequestering C1q in a hostile extracellular environment. In this regard, it has been shown that some *T. cruzi* strains are able to hijack opsonised components of the complement system like MBLs to facilitate their invasion into host cells (Evans-Osses et al. 2014).

Our data also pinpoint the importance of host MBL/ficolin levels to control infection by *T. cruzi*. It is reported that individuals with haplotypes, resulting in lower levels of MBLs, show higher blood parasitaemia during the acute phase of infection, though they may be protected from cardiac damage in the chronic phase of Chagas disease (Luz et al. 2016). In addition, this study shows the value of analysing the expression of multiple parasite complement receptors to explain not only the sensitivity of different *T. cruzi* strains to host complement system, but also their infectivity and virulence properties.
